# Treatment with selective transcatheter arterial embolization of a ruptured profunda artery perforator after internal thigh liposuction: a case report

**DOI:** 10.1186/s13256-023-04067-w

**Published:** 2023-07-26

**Authors:** Pierre Tawa, Tom Boeken, Curtis L. Cetrulo, Alexandre G. Lellouch

**Affiliations:** 1 Vascularized Composite Allotransplantation Laboratory, Center for Transplantation Sciences, Massachusetts General Hospital, Harvard Medical School, Boston, MA USA; 2 Vascular and Oncological Interventional Radiology, University of Paris, Hôpital Européen Georges Pompidou, Paris, France; 3 Department of Plastic, Reconstructive, and Aesthetic Surgery, University of Paris, Hôpital Européen Georges Pompidou, Paris, France

**Keywords:** Liposuction, Bleeding, Hemorrhage, Embolization, Case report

## Abstract

**Background:**

Hemorrhage is an uncommon complication of liposuction that may be trauma-induced by the cannula on the subcutaneous perforators. It usually resolves spontaneously with external compression and results in mild to moderate ecchymosis on the liposuction site. However, in sporadic cases, active bleeding may persist and require urgent intervention for hemostasis.

**Case presentation:**

We report the case of a 60-year-old White female who developed a massive hematoma in the hour following liposuction of the right internal thigh, with active bleeding in the subcutaneous plane reported on contrast-enhanced computed tomography. The initial angiogram was conducted in the right common femoral artery and showed active bleeding from a profunda artery perforator. After careful selective catheterization of the feeding artery using a 2.0-French microcatheter, 0.3 mL of Onyx 34 was injected. Control angiography showed no immediate complication and confirmed the exclusion of the pseudoaneurysm. No postoperative event occurred. Blood pressure and hemoglobin levels remained stable throughout the episode.

**Conclusions:**

Although liposuction is a very common procedure in plastic surgery, hemorrhagic complications may occur and require urgent hemostasis. This case suggests a vital place for interventional radiology in the management of hemorrhagic complications after liposuction.

## Introduction

Since its first description by Illouz [1] in the 1980s, liposuction has become a safe and reliable technique [2, 3] and is now routinely performed. In 2020, over 1,500,000 procedures were reported worldwide, making liposuction the second most frequent procedure in plastic surgery [4]. Today, tumescent liposuction has plenty of indications, including breast reconstruction for autologous fat grafting [5]. However, liposuction is not deprived of complications [6], and potentially life-threatening issues have been reported, including perforation of viscera [7–10], septic shock [9], and hemorrhage [11–13].

Hemorrhage is a relatively uncommon complication that may be trauma-induced by the cannula on the cutaneous perforators during liposuction of the deep subcutaneous layer. It usually resolves spontaneously and results in mild to moderate ecchymosis on the liposuction site. To prevent significant bleeding, tumescent infiltration of the subcutaneous fat layers with a mixture of epinephrine in saline usually precedes liposuction [14]. However, in sporadic cases, active bleeding may persist and require urgent intervention for hemostasis [12, 13]. Herein, we report a case of active hemorrhage after internal thigh liposuction managed with transcatheter embolization.

## Case report

**Fig. 1:**
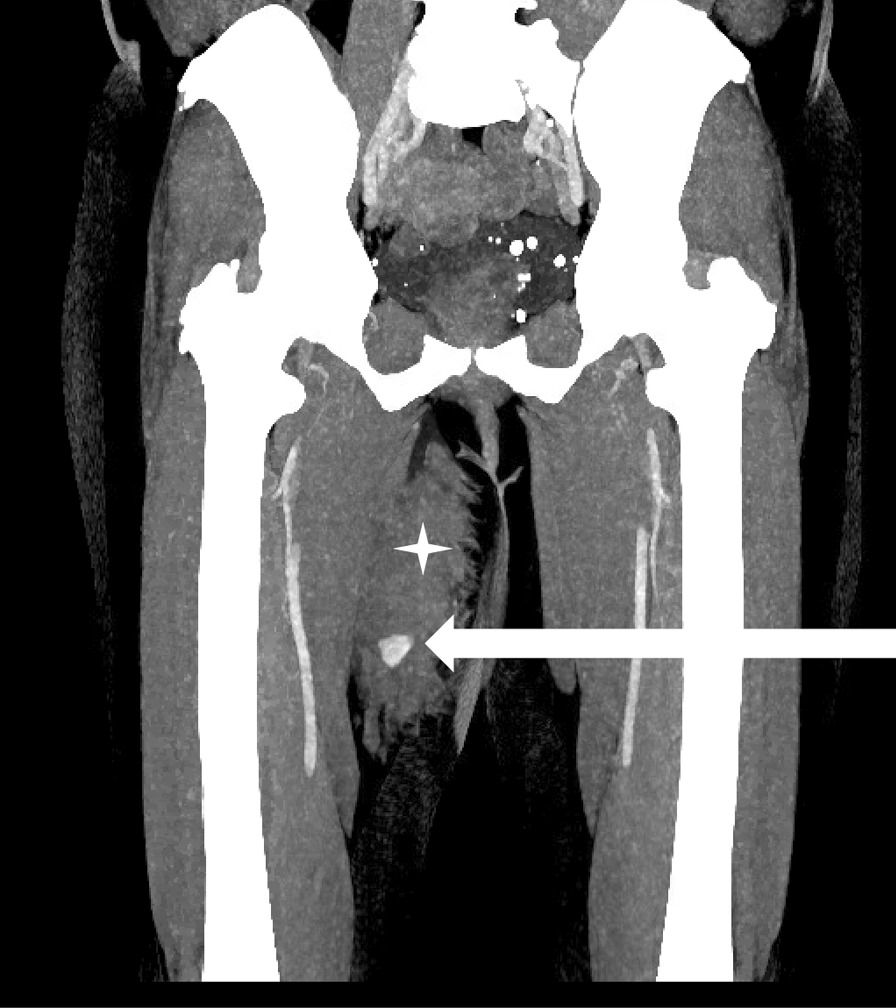
Immediate postoperative contrast-enhanced computed tomography. Coronal view with maximum intensity projection (set at 10 mm) shows a 7-cm hematoma (white star) with a pseudoaneurysm (white arrow). No direct feeding artery is detected

A 60-year-old White woman was admitted as an outpatient for autologous fat transfer as part of her breast reconstruction. She underwent 2 years earlier a left total mastectomy followed by a delayed autologous reconstruction with a deep inferior epigastric perforator (DIEP) free flap performed 6 months ago. Medical history also included treated hypertension and diabetes mellitus. There was no history of abnormal bleeding during previous surgeries. Before liposuction, tumescent infiltration of the subcutaneous fat compartment of the flanks and internal thighs was done with a mixture of 2 mg of epinephrin in a 1-L saline solution. Liposuction was performed with a 3-mm blunt cannula in the deep subcutaneous layer. The total volume of fat harvested was 200 mL per thigh and flank. Fat was processed and filtered through the PUREGRAFT system (Bimini HealthTech, Solana Beach, CA, USA) and reinjected to the left breast. No peroperative event occurred. After 30 min of recovery, the patient complained of pain in the right internal thigh with a rapidly growing mass. A contrast-enhanced computed tomography (CT) scan was performed immediately and showed a voluminous subcutaneous hematoma with active bleeding in contact with the muscle plane (Fig. 1).

**Fig. 2:**
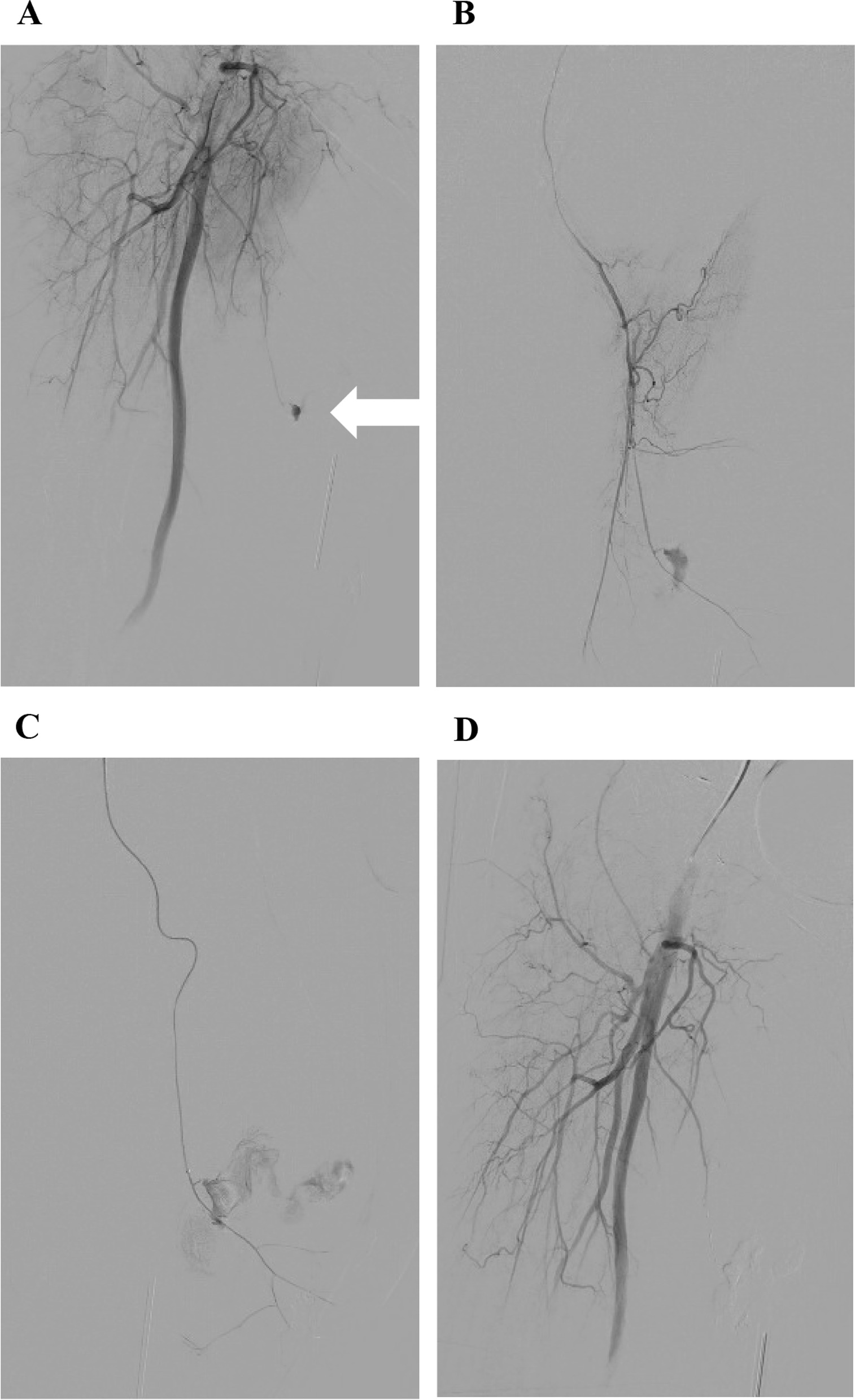
**A** Initial proximal common femoral artery angiography shows active bleeding from a thin profunda artery perforator (white arrow). **B**, **C** Superselective catheterization using a 2.0-French microcatheter shows a proximal (**B**) and distal (**C**) angiography from the feeding artery. Active bleeding is seen and confirms the ruptured pseudoaneurysm. Injection of 0.3 cc of ONYX 34 was performed. **D** Final proximal common femoral artery angiography after injection of Onyx 34 confirms the absence of residual bleeding

**Fig. 3:**
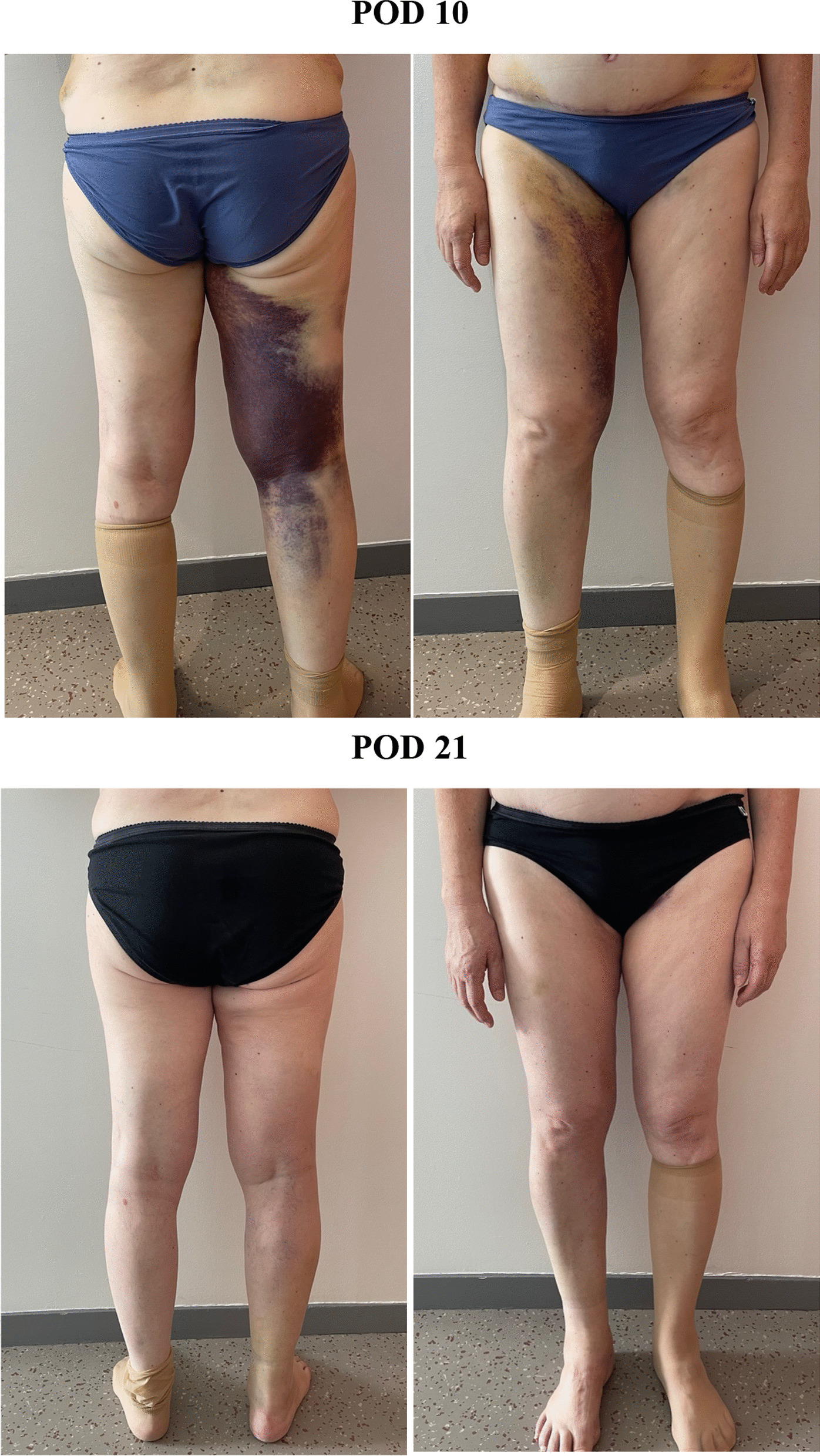
Clinical evolution at postoperative day 10 (above) and postoperative day 21 (below)

The patient was immediately transferred to the interventional radiography department for selective embolization. Access to the left common femoral artery was performed using a 5-French vascular long sheath under local anesthesia. The initial angiogram was conducted in the right common femoral artery and showed active bleeding from a profunda artery perforator (Fig. 2). After careful selective catheterization of the feeding artery using a 2.0-French microcatheter, 0.3 mL of Onyx 34 (Medtronic Inc, Dublin, Ireland) was injected. Control angiography (Fig. 2C) showed no immediate complication and confirmed the exclusion of the pseudoaneurysm. No postoperative event occurred. Blood pressure and hemoglobin levels remained stable throughout the episode. The thigh hematoma was left to spontaneous resorption, and antibiotic prophylaxis with amoxicillin–clavulanate 3 g/day was given for 7 days. The patient was authorized to walk on postoperative day 1, and she was discharged home on postoperative day 2. No further complication occurred, and the hematoma was completely resolved after 3 weeks (Fig. 3).

## Discussion

Liposuction is a standard procedure in reconstructive and cosmetic surgery that is routinely performed on an outpatient basis. The safety and reliability of this surgery have been repeatedly proven in the past decades, with additional benefits of prior tumescent infiltration to ensure optimal anesthesia and bleeding reduction [2, 3, 5]. Despite the use of blunt cannulas, liposuction generates trauma of the subcutaneous vessels and inevitably leads to mild to moderate bleeding in the fat layers. Epinephrin of the tumescent infiltration reduces bleeding, and tranexamic acid can also be used to reduce blood loss, especially in high-volume liposuctions [15, 16]. As described here, massive hemorrhage with active bleeding after liposuction is an infrequent complication with potentially life-threatening consequences if not appropriately addressed [9, 17]. Iatrogenic arterial injuries after orthopedic surgery have been well described [11, 18], and embolization has been shown to be the safest and most effective treatment [19, 20]. However, to our knowledge, only two cases of iatrogenic bleeding after liposuction have been reported in the literature. Choi *et al.* reported a case of active bleeding after abdominal liposuction treated with selective embolization [13]. The 41-year-old patient was admitted to the intensive care unit in hemorrhagic shock 1 day after surgery. The CT scan revealed active bleeding of a branch of the inferior epigastric artery with massive hematoma of the subcutaneous tissues, and immediate selective transcatheter embolization was performed to control the bleeding. In our case, the diagnosis was made prematurely and allowed us to quickly complete the embolization to prevent additional blood loss and acute deglobulation. As in Choi *et al.*’s case, we decided to let the hematoma resolve spontaneously. Evacuation of the hematoma once the bleeding was controlled was not required, as it would add unsightly scars to the internal thigh. In addition, the infectious risk could be easily addressed with antibiotic prophylaxis. Lim *et al.* [12] also reported active bleeding in the abdominal wall after liposuction in a 51-year-old male. Bleeding was controlled by external compression with a large encircling bandage, and the patient was discharged home 3 days after the episode. External compression is an excellent alternative to embolization for nonsurgical hemostasis, although it should be performed with caution in the extremities to prevent compartment syndrome.

## Conclusion

This case highlights the possibility of active bleeding after thigh liposuction requiring a hemostatic intervention. To control such bleeding, we advocate using interventional radiology to perform selective embolization whenever possible. In the absence of interventional radiology facilities, or if the patient’s clinical condition does not allow it, we recommend external compression over the bleeding area. As a last resort, surgical hemostasis should be reserved for refractive cases or if previous options are not feasible due to an unstable patient’s condition.

## Data Availability

Data sharing is not applicable to this article as no datasets were generated or analyzed during the current study.

## Abbreviations

CT: Computed tomography
DIEP: Deep inferior epigastric perforator
